# Further investigation of gateway effects using the PATH study

**DOI:** 10.12688/f1000research.24289.2

**Published:** 2020-11-09

**Authors:** Peter N Lee, John S Fry

**Affiliations:** 1 P.N. Lee Statistics and Computing Ltd., Sutton, Surrey, SM2 5DA, UK; 2 Roe Lee Statistics Ltd., Sutton, Surrey, SM2 5DA, UK

**Keywords:** Cigarettes, Confounding, Over-adjustment, E-cigarettes, Gateway effects, Modelling, Propensity score

## Abstract

**Background: **Interest exists in whether youth e-cigarette use (“vaping”) increases risk of initiating cigarette smoking. Using Waves 1 and 2 of the US PATH study we previously reported adjustment for vaping propensity using Wave 1 variables explained about 80% of the unadjusted relationship. Here data from Waves 1 to 3 are used to avoid over-adjustment if Wave 1 vaping affected variables recorded then.

**Methods:** Main analyses M1 and M2 concerned Wave 2 never smokers who never vaped by Wave 1, linking Wave 2 vaping to Wave 3 smoking initiation, adjusting for predictors of vaping based on Wave 1 data using differing  propensity indices.  M3 was similar but derived the index from Wave 2 data.  Sensitivity analyses excluded Wave 1 other tobacco product users, included other product use as another predictor, or considered propensity for smoking or any tobacco use, not vaping. Alternative analyses used exact age (not previously available) as a confounder not grouped age, attempted residual confounding adjustment by modifying predictor values using data recorded later, or considered interactions with age.

**Results: **In M1, adjustment removed about half the excess OR (i.e. OR–1), the unadjusted OR, 5.60 (95% CI 4.52-6.93), becoming 3.37 (2.65-4.28), 3.11 (2.47-3.92) or 3.27 (2.57-4.16), depending whether adjustment was for propensity as a continuous variable, as quintiles, or the variables making up the propensity score. Many factors had little effect: using grouped or exact age; considering other products; including interactions; or using predictors of smoking or tobacco use rather than vaping. The clearest conclusion was that analyses avoiding over-adjustment explained about half the excess OR, whereas analyses subject to over-adjustment explained about 80%.

**Conclusions: **Although much of the unadjusted gateway effect results from confounding, we provide stronger evidence than previously of some causal effect of vaping, though doubts still remain about the completeness of adjustment.

## Abbreviations

CI, confidence interval; OR, odds ratio; PATH, Population Assessment of Tobacco and Health.

## Introduction

In youths, use of e-cigarettes (“vaping”) has increased considerably in recent years in many countries (e.g. ( Barrington-Trimis *et al.*, 2016; Best *et al.*, 2016; Miech *et al.*, 2019)). It is generally recognized that vaping significantly reduces exposure to harmful constituents compared to smoking ( National Academies of Sciences Engineering and Medicine, 2018), so one might expect risks from vaping to be much lower ( Nutt *et al.*, 2014). However, there are concerns about the rise in vaping. The concern of interest here is the possibility that vaping may encourage some individuals to start smoking who would otherwise not have done so, often referred to as the “gateway” effect. The concern that vaping may act as a gateway into smoking was originally brought sharply into focus by a 2017 meta-analysis ( Soneji *et al.*, 2017) which combined data from nine cohort studies in young people in the US which related previous vaping to later smoking initiation. It reported that, among never-smokers at baseline, ever vaping at baseline strongly predicted initiating smoking in the next 6 to 18 months, with an odds ratio (OR) of 3.62 (95% confidence interval (CI) 2.42-5.41) after adjusting for various factors predictive of initiation. Similarly past 30-day vaping at baseline also predicted later 30-day cigarette use (OR 4.25, 95% CI 2.52-7.37).

We have previously published two papers relating to the gateway effect. Our first paper ( Lee *et al.*, 2018) considered various general issues, including a detailed examination of cohort studies that have reported unadjusted and adjusted estimates of the effect, the nine considered in the 2017 meta-analysis ( Soneji *et al.*, 2017), and six additional studies. It made a number of relevant points:

- The studies that reported that vaping significantly predicts initiation of smoking after adjusting for various other predictors used sets of predictors that were generally quite incomplete.
- Residual confounding arising from the predictors being inaccurately measured was not taken account of in any of the studies.
- Adjusting more precisely may have reduced the association substantially.
- Any true gateway effect would only alter smoking prevalence modestly.
- In youths in the US and UK in 2014–2016 smoking prevalence declined more rapidly than the preceding trend would predict, contrary to what might expect if any large gateway effect existed.
- Even given the existence of some gateway effect, the introduction of e-cigarettes would still likely reduce smoking-related mortality.

We note that a recent meta-analysis ( Khouja *et al.*, 2020) based on 17 studies, 13 considered in our first paper ( Lee *et al.*, 2018) and four more recent studies also pointed to weaknesses in the data, including “reliance on self-report measures of smoking history without biochemical verification”, and noted that the findings did not provide evidence that the “strong consistent association … between e-cigarette use among non-smokers and later smoking” was not due “to shared common causes of both e-cigarette use and smoking”.

Our second paper ( Lee & Fry, 2019) described results of our own analyses, based on data from Waves 1 and 2 of the Population Assessment of Tobacco and Health (PATH) study, a nationally representative longitudinal cohort study in the United States of tobacco use and how it affects the health of people. Wave 1 was conducted from 12 September 2013 to 15 December 2014, with Wave 2 the first annual follow-up. For each Wave, data are available separately for Youths (aged 12–17 years) and Adults (aged 18+ years), the Youth data including some information from the parents. Publicly available data files include extensive information on use of various types of tobacco products and on a range of variables linked to initiation of tobacco. Note that where youths become 18 between successive Waves of the survey, their data will be available in the Adult data rather than the Youth data. Also, additional youths who were under 12 at the time of Wave 1 are added into the Youth data when they reach the age of 12 at a subsequent Wave.

In our main analyses we included youths who had never smoked cigarettes by Wave 1, and had data on smoking initiation by Wave 2. We constructed a propensity score for ever e-cigarette use using variables recorded at Wave 1 and found that adjustment reduced the unadjusted OR markedly, from 5.70 (95% CI 4.33-7.50) to 2.48 (1.85-3.31), 2.47 (1.79-3.42) or 1.85 (1.35-2.53), whether adjustment was made using quintiles of the propensity score, using propensity as a continuous variable, or using each variable making up the score. In sensitivity analyses we confirmed that adjustment explained most of the apparent gateway effect.

Although we found that confounding was a major factor, explaining most of the observed gateway effect, we were particularly concerned about the possibility of over-adjustment, if taking up e-cigarettes had affected the values of some of the Wave 1 predictor variables considered. At the time, we noted that the possibility of over-adjustment could be avoided using data from Waves 1, 2 and 3 of the PATH study, by relating initiation of cigarette smoking at Wave 3 to vaping at Wave 2, restricting attention to those who, at Wave 1, had never vaped, and using propensity indicators recorded at Wave 1 linked to uptake of e-cigarettes by Wave 2.

Here we describe the results of extensive analyses conducted based on Waves 1, 2 and 3. The main objective was to conduct the analyses avoiding the possibility of over-adjustment which was envisaged at the time of our earlier paper ( Lee & Fry, 2019), but we also include a variety of sensitivity and alternative analyses for reasons described below.

## Methods

Some aspects of the analyses described here are the same as those described earlier ( Lee & Fry, 2019) and are not presented again here. The selection of demographic and other predictor variables is as before, except that in some analyses we use exact age (12, 13, 14, 15, 16 and 17), which could now for the first time be estimated from the age group (12 to 14, 15 to 17) at the three Waves and the Wave when youths became adults (18+). Use of the person-level weights provided in the PATH study database is as before, as is the process by which a sequence of logistic regression analyses is used to develop the shorter list of demographic variables to be used in forming the propensity scores.

Our main analysis M1 is the analysis envisaged in our earlier paper ( Lee & Fry, 2019) aimed at avoiding the possibility of over-adjustment in the analyses based only on Waves 1 and 2. It is based on those with data at Waves 1, 2 and 3 who had never smoked cigarettes by Wave 2 and had never used e-cigarettes by Wave 1. This analysis predicts Wave 3 ever smoking from Wave 2 ever e-product use, with adjustment based on Wave 1 predictors used to derive a propensity index for taking up e-products between Waves 1 and 2, and exact age being used in preference to grouped age. Note that, whereas in Wave 1 questions in PATH related only to e-cigarette use, in Waves 2 and 3 questions related to ever e-product use, which also included use of e-cigars, e-pipes and e-hookahs.

As in our earlier paper ( Lee & Fry, 2019) we also conducted four sensitivity analyses (S1 to S4) of analysis M1 which are otherwise similar, except for the following differences:

S1. Those who had ever used other tobacco products at Wave 1 are excluded;

S2. Ever use of other tobacco products at Wave 1 is included as an additional predictor variable;

S3. **T**he analysis is based on a propensity score for ever cigarette smoking rather than for ever vaping; or

S4. The analysis is based on a propensity score for ever use of any tobacco product rather than for ever vaping.

Note that in our original paper ( Lee & Fry, 2019) we also presented results of a further sensitivity analysis, based on linking current vaping to current smoking. This was not repeated here as numbers of new current smokers in current vapers were very low.

Main analysis M2 is similar to M1, except that analysis adjusts for the propensity index as originally derived ( Lee & Fry, 2019), based on 12 variables recorded at Wave 1. This was conducted to gain insight into how critically the estimates of the gateway effect depended on the precise propensity index used. Alternative versions of M2 substitute exact age rather than grouped age in deriving the propensity index, and/or included Wave 1 vapers in the analysis.

Main analysis M3 adjusts for a propensity index derived by linking Wave 2 predictors to Wave 2 e-product use. This is a replicate of the analysis conducted originally ( Lee & Fry, 2019), but using a different period of taking up cigarettes. Data for Wave 1 were ignored, except that where the data for a characteristic was “ever in last 12 months”, Wave 1 data were used to define “ever”. An alternative version of M3 replaces grouped age by exact age in deriving the propensity index.

Apart from analyses linking Wave 2 e-product use to additional cigarette smoking at Wave 3 in those who had never smoked at Wave 2, two additional analyses (A1 and A2) were also conducted.

Additional analysis A1 relates e-cigarette use at Wave 1 to cigarette smoking at Wave 2 as in our earlier publication ( Lee & Fry, 2019), but is based on individuals who provided data at all three Waves. One version of this uses the same 12 variables as before to develop the propensity index, the other replaces grouped age by exact age. The OR from this analysis can be combined with that reported for main analysis M2 to give a combined estimate of the gateway effect for Wave 1 to 2 initiation and Wave 2 to 3 initiation based on the same set of variables determined at Wave 1.

Additional analysis A2 ignores Wave 2 data and relates e-cigarette use at Wave 1 to cigarette smoking at Wave 3 using the same 12 variables as before, but replacing grouped age by exact age.

Consideration of residual confounding was also taken into account for three of the analyses described above (M1, M3, A1), all involving exact age. In each case, the list of predictor variables was unaltered from that used originally, but the values of the predictor variables and of the propensity index were revised based on data available at all three Waves. For age, individual year of age at Wave 1 was used, while gender and Hispanic origin did not change between Waves. For the other variables used to form the propensity index, we used all the available data, generally choosing the response most associated with increased e-cigarette use where response varied between Waves (see Additional File Table 1, *Extended data*, for further details ( Lee, 2020)).

For analyses M1, M3 and A1, alternative versions were also run in which the number of variables adjusted for was increased by also including interactions of age with each of the other three predictors most strongly linked to the relevant gateway effect.

### Software

Relevant data were transferred for analysis to a ROELEE database, and analysed using the ROELEE program (Release 59, Build 49). All these analyses could be run using the GLM Package and the Step Function from the R Program ( https://www.r-project.org/).

## Results

### M1: Relating initiation of cigarette smoking between Waves 2 and 3 to ever e-product use at Wave 2, with adjustment for Wave 1 predictors linked to uptake of e-cigarettes between Waves 1 and 2

Initial analyses linked exact age, four other demographic variables (gender, Hispanic origin, race and census region) and 60 other selected predictor variables to ever e-product use at Wave 2 in those who had not smoked or used e-cigarettes at Wave 1. A propensity index based on 16 variables was derived using the three step process described earlier ( Lee & Fry, 2019). Additional File Table 2 (see *Extended data* ( Lee, 2020)) shows the steps at which different variables were eliminated from consideration, while Table 1 gives the fitted equation for the propensity index.

**Table 1.** Predicting Wave 2 ever e-product use from 16 Wave 1 predictor variables (Main analysis M1).

Variable ^a^	Levels	N	OR (95% CI)
Exact age	12	1518	1.00 (base)
	13	1474	1.71 (1.23-2.38)
	14	1451	1.97 (1.43-2.71)
	15	1376	2.25 (1.65-3.08)
	16	1188	2.55 (1.86-3.51)
	17	1051	3.75 (2.72-5.15)
Ever been curious about smoking a cigarette			0.86 (0.76–0.97) ^b^
Think you will smoke a cigarette in the next year			0.59 (0.48–0.71) ^c^
Anyone who lives with you now use tobacco	Cigarettes, cigars, cigarillos, filtered cigars	2140	1.00 (base)
	Smokeless or other tobacco only	319	1.73 (1.26-2.37)
	No-one living in the home uses tobacco	5599	0.78 (0.65-0.94)
Ever used alcohol at all	Yes	2483	1.00 (base)
	No	5575	0.53 (0.45-0.62)
Agree/disagree: like new and exciting experiences, even if I have to break the rules	Strongly agree	285	1.00 (base)
	Agree	1252	0.71 (0.52-0.97)
	Neither agree nor disagree	2107	0.64 (0.47-0.87)
	Disagree	2404	0.38 (0.28-0.53)
	Strongly disagree	2010	0.46 (0.32-0.65)
Youth’s grade performance in school in past 12 months	Mostly A’s	2342	1.00 (base)
	A’s or B’s	2849	1.30 (1.07-1.58)
	Mostly B’s	702	1.60 (1.22–2.10)
	B’s or C’s	1346	1.47 (1.17–1.85)
	Mostly C’s	325	2.16 (1.52-3.09)
	C’s or D’s	334	2.74 (1.95-3.86)
	Mostly D’s	45	2.09 (0.90-4.87)
	D’s or F’s	71	2.54 (1.34-4.81)
	Mostly F’s	10	1.85 (0.26-12.91)
	School is ungraded	34	1.80 (0.54-6.06)
How often you visit your Facebook, Google Plus, MySpace, Twitter or other	Several times a day	2464	1.00 (base)
	About once a day	2284	0.67 (0.56-0.80)
	3–5 days a week	1006	0.73 (0.58-0.92)
	1–2 days a week	732	0.51 (0.37-0.69)
	Never	1572	0.40 (0.31-0.53)
Agree/disagree: I think I would enjoy using tobacco	Strongly agree	18	1.00 (base)
	Agree	95	0.42 (0.14–1.31)
	Disagree	1517	0.57 (0.20-1.58)
	Strongly disagree	6428	0.35 (0.12-1.01)
Hispanic origin	Hispanic	2332	1.00 (base)
	Not Hispanic	5726	0.67 (0.57-0.79)
Became very distressed when something reminded of past	Past month	1940	1.00 (base)
	2–12 months	1137	0.86 (0.70–1.07)
	Over a year	906	0.71 (0.55-0.92)
	Never	4075	0.74 (0.62-0.89)
Cigarettes or tobacco might be available to youth at parent or guardian’s home	Yes	1057	1.00 (base)
	No	7001	0.65 (0.52-0.80)
Money received in total during an average week	None	2771	1.00 (base)
	Less than $1	331	1.34 (0.91-1.96)
	$1 to $5	1234	1.26 (0.99-1.61)
	$6 to $10	1019	1.40 (1.10-1.79)
	$11 to $20	1289	1.42 (1.14-1.77)
	$21 to $50	751	1.36 (1.06-1.75)
	$51 to $100	337	1.53 (1.11-2.10)
	$101 to $150	160	2.02 (1.33-3.06)
	$151 or more	166	1.96 (1.29-2.99)
Last time 2+ times: had a hard time paying attention at school, work or home	Past month	2700	1.00 (base)
	2–12 months	1402	0.75 (0.62-0.92)
	Over a year	819	0.84 (0.64-1.09)
	Never	3137	0.72 (0.59-0.87)
Number of times seen Movie 4	Never	6839	1.00 (base)
	Once	858	0.91 (0.73-1.11)
	Twice	190	1.24 (0.83-1.86)
	3 or more times	171	1.91 (1.29-2.82)
Think you will try a cigarette soon			1.99 (1.17-3.37) ^d^

Note: The model is based on 8058 youths with data on all 16 predictors who neither smoked nor used e-cigarettes at Wave 1. ^a^ The variables are shown in order of their inclusion into the model. ^b^ The OR is per unit of the graded variable which represents decreasing curiosity. ^c^ The OR is per unit of the graded variable which represents decreasing likelihood. ^d^ The OR is per unit of the graded variable which represents decreasing likelihood, with those originally entered as missing because they thought that they would not smoke a cigarette in the next year scored as “definitely not” (Level 4).

As shown in Table 2, adjustment for propensity removed about half the excess OR (i.e. OR−1), the unadjusted OR of 5.60 (95% CI 4.52-6.93) reducing to either 3.37 (2.65-4.28) or 3.11 (2.47-3.92), depending on whether adjustment was as a continuous variable or as quintiles. A similar reduction in the OR, to 3.27 (2.57-4.16), was achieved by adjusting for the 16 variables individually. It can also be seen that, for the first seven variables adjusted for, the adjusted OR decreased steadily, to 3.25. Further adjustment had little or no effect, with introducing additional variables sometimes slightly increasing the estimated OR and sometimes slightly decreasing it.

**Table 2.** Relating Wave 3 ever smoking to Wave 2 ever e-product use (Main analysis M1).

Adjustment variables	OR (95% CI)
None	5.60 (4.52-6.93)
Propensity score as quintiles	3.11 (2.47-3.92)
Propensity score as a continuous variable	3.37 (2.65-4.28)
Exact age	4.87 (3.91-6.06)
+ Ever been curious about smoking a cigarette	4.27 (3.41-5.34)
+ Think you will smoke a cigarette in the next year	3.84 (3.06-4.82)
+ Anyone who lives with you now use tobacco	3.73 (2.97-4.69)
+ Ever used alcohol at all	3.48 (2.76-4.38)
+ Agree/disagree: Like new and exciting experiences even if I have to break the rules	3.39 (2.68-4.28)
+ Youth’s grade performance in school in past 12 months	3.25 (2.57-4.12)
+ How often you visit your Facebook, Google Plus, MySpace, Twitter or other	3.17 (2.50-4.01)
+ I think I would enjoy using tobacco	3.17 (2.50-4.02)
+ Hispanic origin	3.22 (2.54-4.09)
+ Last time a significant problem with: becoming very distressed when something reminded of past	3.19 (2.51-4.05)
+ Cigarettes or tobacco might be available to youth at parent or guardian’s home	3.17 (2.50-4.02)
+ Money received in total during an average week	3.25 (2.56-4.13)
+ Last time 2+ times: Had a hard time paying attention at school, work or home	3.22 (2.53-4.09)
+ Number of times seen Movie 4	3.28 (2.57-4.17)
+ Think you will try a cigarette soon	3.27 (2.57-4.16)

Notes: The table shows the effects of adjustment based on the Wave 1 predictors used to derive a propensity index for taking up e-products between Wave 1 and 2. The analyses are based on those with data at Waves 1, 2 and 3 who had never smoked cigarettes by Wave 2 and had never used e-cigarettes by Wave 1. Between Waves 2 and 3 261/7367 (3.54%) of never users of e-products at Wave 2 took up smoking, while 148/893 (16.57%) of ever users did so. For individuals who were 16 or 17 at Wave 1, adult data were used to determine e-product use and cigarette smoking at later Waves. The table includes the results of a stepwise regression based on successively including the most significant adjustment variables, given that ever e-product use at Wave 2 was included in the model.

Four sensitivity analyses of M1 were carried out, fuller details being given in Table 3 to Table 6 of the Additional File (see *Extended data* ( Lee, 2020)).

Compared to M1, S1 excluded those who had ever used products other than cigarettes or e-cigarettes at Wave 1, both in the construction of the propensity index and in estimating the gateway effect. Whereas M1 involved 8260 youths, of which 409 initiated smoking between Waves 2 and 3, S1 involved 7945, of which 359 took up smoking. The propensity index developed for S1 involved all the 16 variables shown in Table 2, except for “Number of times seen Movie 4” and “Think you will try a cigarette soon”. Here, the pattern of results is similar to that for Table 2, with the unadjusted OR of 5.66 (95% CI 4.49-7.13) reducing to either 3.45 (2.67–4.46), 3.24 (2.53–4.15), or 3.23 (2.49–4.18), depending on whether adjustment was made for propensity as a continuous variable, propensity as quintiles, or all the 14 variables individually.

Compared to M1, the only difference for S2 was that ever smoked other tobacco products at Wave 1 was added to the 16 variables used in M1 to make up the propensity score, and was forced into the regression models. Starting with the same unadjusted OR as M1, the adjusted ORs were very similar; 3.37 (2.64–4.29), 3.07 (2.44-3.87) and 3.20 (2.50-4.08), after adjustment for propensity (continuous), propensity (quintiles), or all the individual variables.

Whereas M1 (and S1 and S2) adjusted for variables found to be predictive of initiating e-product use at Wave 2, S3 adjusted for variables predictive of cigarette smoking. Here, the final model included 27 variables. The unadjusted OR of 5.65 (95% CI 4.55-7.01) slightly differed from that in M1 as the individuals considered had to have non-missing data on 27 variables rather than 16. However, the overall effect of adjustment was again similar, with the OR reducing to 3.28 (2.56-4.22) after adjustment for all 27 variables. As for M1, adjustment for the first four variables had the most effect. Adjustment for the first seven variables reduced the OR to 3.26 (2.57-4.13), similar to the OR after adjustment for all 27. Propensity adjustment was not carried out in S3.

Compared to M1, S4 adjusted for variables predictive of take-up of any tobacco product between Waves 1 and 2. Here, the propensity index was based on 18 variables, with the unadjusted OR of 5.74 (4.55-7.23) reducing to 3.31 (95% CI 2.56-4.28), 3.19 (2.48-4.09), or 3.21 (2.47-4.18), after adjustment for propensity (continuous), propensity (quintiles), or all the individual variables. Adjustment for all 18 variables had a similar effect to adjustment for the most important 10 variables, where the OR was 3.20 (2.47-4.14).

### M2: Relating initiation of cigarette smoking between Waves 2 and 3 to ever e-product use at Wave 2, with adjustment for the same Wave 1 predictors as previously reported ( Lee & Fry, 2019)

Here, instead of deriving the Wave 1 predictors linked to uptake of e-cigarettes between Waves 1 and 2, analysis M2 uses the same set of Wave 1 predictors used in our earlier work ( Lee & Fry, 2019), the results being shown in Table 3. Here, the unadjusted OR of 5.74 (95% CI 4.62-7.13) reduced to 3.54 (2.81-4.45) after adjustment for propensity as quintiles and to 3.45 (2.72-4.37) after adjusting for the individual variables. While adjustment here removed about half the excess OR, the reduction was less, to 4.53 (3.62-5.68), after adjustment for propensity as a continuous variable. The reductions were similar if exact age rather than age group was included in the list of variables. Here, the unadjusted OR was reduced to 3.51 (2.79-4.41) after adjustment for propensity as quintiles, 4.59 (3.66-5.74) after adjustment for propensity as a continuous variable, and 3.39 (2.67-4.30) after adjustment for the individual variables.

**Table 3.** Relating Wave 3 ever smoking to Wave 2 ever e-product use (Main analysis M2).

Adjustment variables		OR (95% CI)
None		5.74 (4.62-7.13)
Propensity score as quintiles		3.54 (2.81-4.45)
Propensity score as continuous variable		4.53 (3.62-5.68)
Age range		5.20 (4.17-6.49)
+	Ever used alcohol at all	4.45 (3.54-5.58)
+	Ever been curious about smoking a cigarette	4.10 (3.26-5.16)
+	Think you will smoke a cigarette in the next year	3.70 (2.94-4.68)
+	Agree/disagree: Prefer friends who are exciting and unpredictable	3.65 (2.89-4.61)
+	Reaction if parent/guardian found you using tobacco	3.64 (2.88-4.60)
+	Gender	3.63 (2.87-4.58)
+	Agree/disagree: I think I would enjoy using tobacco	3.63 (2.87-4.59)
+	Agree/disagree: Some products are safer than others	3.63 (2.87-4.59)
+	Ever used prescription drug not prescribed to you: Ritalin or Adderall	3.67 (2.90-4.64)
+	Has a Facebook, Google Plus, MySpace, Twitter or other social networking	3.53 (2.79-4.47)
+	Anyone who lives with you now use tobacco	3.45 (2.72-4.37)

Notes: The table shows the effects of adjustment based on the same Wave 1 predictors as used in our original paper ( Lee & Fry, 2019). The analyses are based on those with data at Waves 1, 2 and 3 who had never smoked cigarettes by Wave 2 and had never used e-cigarettes by Wave 1. Between Waves 2 and 3, 249/7133 (3.49%) of never users of e-products at Wave 2 took up smoking, while 146/880 (16.59%) of ever users did so. For individuals who were 16-17 at Wave 1, adult data were used to determine e-product use and cigarette smoking at later Waves. The table includes the results of a stepwise regression based on successively including the most significant adjustment variables, given that ever e-product use at Wave 2 was included in the model.

Similar analyses were also run that did not exclude those who had used e-cigarettes by Wave 1. This increased the number of ever e-product users who took up smoking from 146 to 201, and slightly increased the unadjusted OR to 5.95 (4.89-7.23). However, the pattern of decline following adjustment was quite similar. For example, the OR adjusted for the individual variables reduced to 3.31 (2.65-4.12) using grouped age and to 3.26 (2.62-4.06) using exact age.

### M3: Relating initiation of cigarette smoking between Waves 2 and 3 to ever e-product use at Wave 3, with adjustment for Wave 2 predictors

As noted in the Methods section, M3 is essentially a replicate of our earlier work ( Lee & Fry, 2019), but using a different period of introduction of cigarettes. The propensity score developed was based on 18 variables, using age group or exact age as alternatives. The results, shown in Table 4, indicate that, as earlier ( Lee & Fry, 2019), a large proportion of the unadjusted association can be explained by adjustment. The largest proportion was explained by adjusting for the 18 variables making up the propensity score, with the unadjusted OR of 6.70 (95% CI 5.40-8.32) reducing to 2.25 (1.74-2.91) or 2.75 (1.75-2.93) depending on whether the list of variables included age range or exact age. However, most of this reduction could be explained by adjustment for propensity.

**Table 4.** Relating Wave 3 ever smoking to Wave 2 ever e-product use (Main analysis M3).

Adjustment variables	Using age group OR (95% CI)	Using exact age OR (95% CI)
None	6.70 (5.40-8.32)	6.70 (5.40-8.32)
Propensity score as quintiles	2.77 (2.19-3.50)	2.74 (2.17-3.48)
Propensity score as a continuous variable	2.57 (1.98-3.33)	2.60 (2.00-3.36)
Age range	5.78 (4.62-7.22)	-
Exact age	-	5.45 (4.36-6.83)
+ Last time a significant problem with: feeling very trapped, lonely, sad, blue, depressed	5.22 (4.17-6.54)	4.95 (3.94-6.21)
+ Reaction if parent/guardian found you using tobacco	4.89 (3.89-6.14)	4.66 (3.70-5.87)
+ Money received in total during an average week	4.65 (3.69-5.86)	4.52 (3.59-5.71)
+ Number of times seen Movie 3	4.31 (3.41-5.44)	4.20 (3.32-5.31)
+ Number of times seen Movie 4	4.12 (3.25-5.21)	4.02 (3.18-5.10)
+ Ever been curious about smoking a cigarette	3.45 (2.71-4.38)	3.36 (2.64-4.28)
+ Think you will smoke a cigarette in the next year	2.89 (2.26-3.70)	2.86 (2.24-3.66)
+ Ever used alcohol at all	2.63 (2.05-3.37)	2.63 (2.05-3.38)
+ In past 12 months, youth’s grade performance at school	2.51 (1.95-3.22)	2.51 (1.95-3.23)
+ Agree/disagree: using tobacco would help me calm down when I am angry	2.43 (1.89-3.12)	2.43 (1.89-3.13)
+ How often you visit your social media accounts	2.43 (1.88-3.12)	2.45 (1.90-3.15)
+ Would smoke if one of your friends offered you one	2.37 (1.84-3.06)	2.39 (1.86-3.09)
+ Anyone who lives with you now use tobacco	2.34 (1.81-3.02)	2.36 (1.83-3.04)
+ Think you will try a cigarette soon	2.33 (1.81-3.01)	2.35 (1.82-3.03)
+ Agree disagree: some tobacco products are safer than others	2.30 (1.78-2.97)	2.32 (1.79-2.99)
+ Youth has a curfew or set time to be home on school nights	2.29 (1.77-2.95)	2.30 (1.78-2.98)
+ Ever used prescription drug not prescribed to you: Ritalin or Adderall	2.25 (1.74-2.91)	2.27 (1.75-2.93)

Notes: The table shows the effects of adjustment based on Wave 2 predictors linked to use of e-products in Wave 2. The analyses are based on those with data at Waves 2 and 3 ignoring data from Wave 1. Between Waves 2 and 3, 228/8233 (2.77%) of never users of e-products at Wave 2 took up smoking, while 145/949 (15.28%) of ever users did so. For individuals who were 17 at Wave 2, adult data were used to determine cigarette smoking at Wave 3. The table includes the results of a stepwise regression based on successively including the most significant adjustment variables, given that ever e-product use at Wave 2 was included in the model. The first set of ORs is based on a model including age group, while the second is based on a model including exact age.

Combining the Wave 2 to 3 results shown in Table 4 with the Wave 1 to 2 results reported earlier ( Lee & Fry, 2019) by fixed-effect meta-analysis gives an unadjusted OR of 6.30 (5.31-7.46), which is reduced to 2.65 (2.24-3.18), 2.53 (2.07-3.10) or 2.08 (1.70-2.54) depending on whether adjustment is for propensity (quintiles), propensity (continuous) or all the variables making up the propensity score. This represents reductions in the excess OR of, respectively, 68.9%, 71.1% or 79.8%.

### A1: Relating initiation of cigarette smoking between Waves 1 and 2 to ever e-cigarette use at Wave 1, based on individuals who provided data at all three Waves

Table 5 summarizes the main results of these analyses and compares them with those reported earlier ( Lee & Fry, 2019). While the original analyses were based on 9423 youths, 421 of whom initiated smoking, the new analyses were based on 8700 youths, 389 of whom initiated smoking. As can be seen, the results in the original analysis, based on grouped age, were similar to those from the new analyses, whether grouped or exact age was used.

**Table 5.** Relating Wave 2 ever smoking to Wave 1 ever e-cigarette use - original ( Lee & Fry, 2019) and A1 ORs.

Adjustment variables	Data on two Waves	Data on all three Waves	
	Originally reported OR (95% CI)	Grouped age OR (95% CI)	Exact age OR (95% CI)
None	5.70 (4.33-7.50)	5.99 (4.52-7.95)	5.99 (4.52-7.95)
Propensity score as quintiles	2.48 (1.85-3.31)	2.65 (1.96-3.58)	2.59 (1.92-3.50)
Propensity score as continuous variable	2.47 (1.79-3.42)	2.67 (1.92-3.72)	2.64 (1.89-3.68)
Grouped age	4.81 (3.64-6.35)	5.04 (3.78-6.72)	-
Exact age	-	-	4.81 (3.60-6.42)
+11 further variables	1.85 (1.35-2.53)	1.97 (1.42-2.73)	1.98 (1.43-2.75)

Notes: Each set of ORs is based on those who had never smoked cigarettes by Wave 1. The first analysis is as summarized in Table 1. The last two analyses only exclude those without data at Wave 3.

The results from analysis A1 for grouped age may theoretically be combined with those from analysis M2 shown in Table 3, as they both use the Wave 1 predictors from our original paper ( Lee & Fry, 2019), with exact age replacing grouped age, and are both based on individuals with data at all three Waves. However, as illustrated by the results adjusted for all 12 variables, where the ORs are 3.45 (95% CI 2.72-4.37) from Table 3 and 1.97 (1.42-2.73) from Table 5, these estimates are heterogeneous (p<0.001), providing a random-effects combined estimate of 2.64 (1.52-4.57).

### A2: Relating Wave 3 ever smoking to Wave 1 e-cigarette use, ignoring Wave 2 data

This analysis is similar to that reported originally ( Lee & Fry, 2019) but relates to a longer follow-up period, and uses exact rather than grouped age. The results of this analysis, shown in Table 6, are quite similar to those shown in Table 5. Again, an unadjusted OR is markedly reduced by adjusting for propensity, whether as quintiles or as a continuous variable, and is further reduced by adjusting for all the 12 individual variables considered.

**Table 6.** Relating Wave 3 ever smoking to Wave 1 ever e-cigarette use using exact age.

Adjustment variables		OR (95% CI)
None		5.65 (4.50-7.10)
Propensity score as quintiles		2.48 (1.95-3.16)
Propensity score as continuous variable		2.61 (2.00-3.40)
Exact age		4.69 (3.71-5.93)
+	11 further variables	1.97 (1.51-2.56)

Notes: The table shows the effects of adjustment based on the same Wave 1 predictors as used in our original paper ( Lee & Fry, 2019) but replacing age range by exact age. The set of ORs is based on those with data at Waves 1, 2 and 3 who had never smoked cigarettes by Wave 1. Between Waves 1 and 3, 716/8334 (8.59%) of never users of e-cigarettes at Wave 1 took up smoking, while 123/366 (33.61%) of ever users did so. The table includes the results of a stepwise regression based on successively including the most significant adjustment variables, given that ever e-product use at Wave 1 was included in the model.

### Attempting to account for residual confounding

Table 7 summarizes the main results shown in Table 2 for main analysis M1, which make no allowance for residual confounding, and compares them with the results of an analysis using the same list of predictor variables, but with values modified in an attempt to adjust for residual confounding. As can be seen, markedly more of the unadjusted association was explained when allowance for residual confounding was made, with the adjusted ORs in the range 2.36 to 2.46 when allowance was made, compared with 3.11 to 3.37 when it was not. Note that the unadjusted ORs in the two sets of results vary slightly, as missing values in some individuals in the original analyses were replaced by estimates taken from other Waves.

**Table 7.** Effect of allowance for residual confounding in main analysis M1.

Adjustment variables	M1 – no allowance OR (95% CI)	M1 – allowance OR (95% CI)
None	5.60 (4.52-6.93)	5.65 (4.58-6.98)
Propensity score as quintiles	3.11 (2.47-3.92)	2.40 (1.91-3.02)
Propensity score as a continuous variable	3.37 (2.65-4.28)	2.46 (1.93-3.14)
All 16 variables individually	3.27 (2.57-4.16)	2.36 (1.85-3.02)

Notes: The “no allowance” results correspond to those in Table 6. The analyses are based on those with data at Waves 1, 2 and 3 who had never smoked cigarettes by Wave 2 and had never used e-cigarettes by Wave 1. Between Waves 2 and 3 261/7367 (3.54%) of never users of e-products at Wave 2 took up smoking, while 148/893 (16.57%) of ever users did so in the population considered in the “no allowance” analyses The corresponding figures in the “allowance” analyses were 267/7682 (3.48%) and 150/915 (16.39%). For individuals who were 16 or 17 at Wave 1, adult data were used to determine e-product use and cigarette smoking at later Waves. The table includes the results of a stepwise regression based on successively including the most significant adjustment variables, given that ever e-product use at Wave 2 was included in the model.

While allowance for residual confounding has quite a marked effect for analysis M1, the analysis which avoided the possibility of over-adjustment, it did not for analyses M3 and A2, which did not avoid this possibility. Detailed results are shown in Table 7 and Table 8 in the Additional File (see *Extended data* ( Lee, 2020)).

**Table 8.** Summary of results from analyses.

		Baseline	Follow-up				Unadjusted	% Excess OR explained ^a^			
	Analysis	Wave	Wave	Predictor	Age	Comment	OR	P as Q ^b^	P as C ^c^	6 variables	All variables
A	Original	1	2	Ever e-cigs	Grouped	As published ( ( Lee & Fry, 2019))	5.70	68.5	68.7	78.1	81.9
B	M1	2	3	Ever e-cigs	Exact	Predictors revised based on those who were not Wave 1 e-users	5.60	54.1	48.5	48.0	50.7
C	M1/S1	2	3	Ever e-cigs	Exact	As M1 but excludes Wave 1 other product users	5.66	51.9	47.4	47.9	52.1
D	M1/S2	2	3	Ever e-cigs	Exact	As M1 but Wave 1 other product use included as predictor	5.60	55.0	48.3	50.2	52.2
E	M1/S3	2	3	Ever cigs	Exact	As M1 but adjusting for predictors of ever cigarette smoking	5.65	-	-	48.4	51.0
F	M1/S4	2	3	Ever any product	Exact	As M1 but adjusting for predictors of ever any tobacco use	5.74	53.8	51.3	45.1	53.4
G	M2	2	3	Ever e-cigs	Grouped	Original 12 predictors	5.74	46.4	25.5	44.3	48.3
H	M2 (variant)					Did not exclude Wave 1 e-users	5.95	50.3	28.7	49.3	53.3
I	M2 (variant)	2	3	Ever e-cigs	Exact	Original 12 predictors	5.74	47.0	24.3	46.0	49.6
J	M2 (variant)					Did not exclude Wave 1 e-users	5.95	50.7	28.3	50.5	54.3
K	M3	2	3	Ever e-cigs	Grouped	Predictors revised essentially ignoring Wave 1 data	6.70	68.9	72.5	45.3	78.1
L	M3 (variant)				Exact	As above but using exact age	6.70	69.5	71.9	47.0	77.8
M	A1	1	2	Ever e-cigs	Grouped	As original but based on those with data on all three Waves	5.99	66.9	66.5	76.8	80.6
N	A1 (variant)				Exact	As above but using exact age	5.99	68.1	67.1	77.0	80.4
O	A2	1	3	Ever e-cigs	Exact	Original predictors but ignoring Wave 2	5.65	68.2	65.4	74.4	79.1
P	M1 (variant)	2	3	Ever-e-cigs	Exact	As M1 but allows for residual confounding	5.65	69.9	68.6	60.0	70.8
Q	M3 (variant)	2	3	Ever e-cigs	Exact	As M3 but allows for residual confounding	6.67	75.3	74.3	51.5	80.2
R	A1 (variant)	1	2	Ever e-cigs	Exact	As A1 but allows for residual confounding	6.10	69.0	68.0	65.1	76.7

^a^ % excess explained =100*(OR _u _– OR _A_) / (OR _u_–1) where OR _u_ is the unadjusted OR, and OR _A_ is the adjusted OR. ^b^ P as Q = propensity as quintiles. ^c^ P as C = propensity as a continuous variable.

### Investigating whether introducing some interactions explains more of the gateway effect

Versions of analyses M1, M3 and A1 were also seen, in which the number of variables adjusted for was extended by also including interactions of age with each of the other three predictors most strongly linked to the gateway effect. For analysis M1, allowance for these interactions had virtually no effect, the original estimate of 3.27 (95% CI 2.57-4.16) shown in Table 2 without including interactions changing to 3.26 (2.55-4.15) when interactions were included in the model. For analysis M3, the estimate changed only from 2.27 (1.75-2.93) to 2.35 (1.81-3.05), while for analysis A1, it changed from 1.98 (1.43-2.75) to 2.06 (1.48-2.88).

### Summary of results

Table 8 summarizes the results from 18 of the analyses described above, expressing the extent to which adjustment explained the unadjusted OR using the statistic 100 x (OR _U_ – OR _A_) / (OR _U_ – 1) where OR _U_ is the unadjusted OR, and OR _A_ is the adjusted OR. The most obvious impression from the table is that the results largely fall into two groups.

Results from the original analysis and for analyses M3, A1 and A2 (rows A, K to O, and Q to R of Table 8) all show that as much as about 80% of the unadjusted excess OR can be explained by adjustment for the full set of variables in the model, with somewhat less, typically about 70%, explained using propensity as quintiles or as a continuous variable.

In contrast, results from virtually all of analyses M1 and M2 (rows B to K) show that only about 50% of the unadjusted excess OR can be explained by adjustment for the full set of variables, with propensity as quintiles giving generally similar results.

The difference between these two groups is that the first set of results are subject to the problem of over-adjustment, with the values of the predictors used possibly having been affected by having used e-cigarettes. This is mainly so where the baseline Wave was Wave 1, but was also true for analysis M3 where Wave 1 data were essentially ignored. In contrast, the second set of results avoided over-adjustment by considering follow-up from Wave 2 to 3, with predictors based on Wave 1 data in youths who had never used e-cigarettes. However, in this second set of results the variables used were not as up-to-date as in the first analyses.

The variant analysis of M1, allowing for residual confounding (row P), gives an intermediate result, with about 70% of the excess risk being explained, whether by the full set of variables or by propensity. This analysis, however, does not avoid the problem of over-adjustment as it incorporates some information from Waves where individuals were already using e-cigarettes.

It is clear from Table 8 that many of the variables studied had little effect on the pattern of results. These included use of grouped or exact age, taking into account use of other products, and using predictors of cigarette smoking or any tobacco use rather than predictors of e-cigarette use.

Two other conclusions may be drawn from Table 8. One is that adjustment for propensity as quintiles or as a continuous variable generally gives very similar results, with the exception of analysis M2 and its variants, where propensity as a continuous variable explained substantially less of the unadjusted excess OR. Inspection of the detailed modelling results showed that, whereas in other analyses, the logarithm of the OR increased fairly linearly with quintiles of propensity, in the case of analysis M2 and its variants it did not. Thus, in M1 for example, the log ORs by quintile were 0, 0.73, 1.11, 1.66 and 2.52, while in M2 they were 0, 0.21, 0.96, 1.51 and 2.19, with very little rise between quintiles 1 and 2.

The other is that adjustment for the first six variables in the model generally explained a very substantial part of the unadjusted excess OR explained by the full set. Though this was not true for analysis M2, it was still true that adjustment for the last eight or nine variables explained far less of the excess OR than did the first eight or nine.

## Discussion

In our publication based on Waves 1 and 2 ( Lee & Fry, 2019) our analyses showed that an unadjusted estimate of the gateway effect 5.70 (85% CI 4.33-7.50) could be considerably reduced by adjustment, to 1.59 (1.14-2.20) in the most striking case. Because of the marked reduction in the OR following adjustment, and the possibility of incomplete control for confounding we regarded it as “unclear whether prior vaping actually increases uptake of cigarette smoking”. However, we did note the possibility of over-adjustment, with vaping at Wave 1 possibly having affected the recorded values of some of the variables used for adjustment.

At that time we noted that this possibility of over-adjustment could be addressed in analyses relating initiation of cigarette smoking at Wave 3 to vaping at Wave 2, restricting attention to those youths who, at Wave 1, had never vaped, and using adjustment variables recorded at Wave 1. This we have done in the analyses reported here, and our major finding is that adjustment reduced the excess risk far less, by only about 50% rather than about 80%, in our main analysis M1.

While these results more strongly support the existence of a true gateway effect of taking up vaping, there must still remain doubt about its magnitude. One reason is that predictors recorded a year before the baseline may not fully account for the characteristics of the youth at the start of follow-up. A second reason is that, although the PATH study records data on a whole range of possibly relevant characteristics, there may be some relevant predictors or interactions of predictors not considered. A third reason is that the answers to some of the questions may have been inaccurately measured. We have attempted to address this problem of residual confounding by amending values of predictors recorded at Wave 1 to take into account data recorded at later Waves. However, this problem re-introduces the problem of over-adjustment as Wave 2 and 3 values may have been affected by vaping. Theoretically, one could use data from Waves 1 to 4, using data for Waves 1 and 2 from youths who have never vaped to produce more accurate estimates of the predictors to use for a study of gateway effects between Waves 3 and 4. But this would add to the problem of using predictors recorded some time before follow-up.

Other issues are possible biases arising due to loss to follow-up and missing data. To address this in relation to our main analysis M1, we compared the distribution of the demographic variables age (at Wave 2), sex, Hispanic origin, race and census region between (A) the 8260 youths considered in Table 2, (B) the 716 for whom no data on cigarettes were available at Wave 3 (due mainly to lack of follow-up but partly to missing responses at Wave 3), and (C) the 537 for whom data on cigarettes at Wave 3 were available, but data were missing on one or more of the 16 predictors making up the propensity score. Compared to youths in group A, those in group B were somewhat more often White (weighted percentages 70.0 in A, 74.6 in B) and older (43.8% age 15-17 in A, 48,1% in B), but were otherwise very similar. Again compared to group A, those in group C were somewhat more likely to be Black (15.5% in A, 22.2% in C) and were clearly younger (56.2% age 12-14 in A, 70.7% in C). Again, little difference was seen in regard to sex, Hispanic origin or census origin. Given the overall loss of youths for whom results might have been available (1253/9513 = 13.1%) is not large, the generally quite small between-group differences seen, the lack of evidence of any interaction of age with other major predictors, and the fact that race did not feature in the derived propensity index, it seems unlikely to us that any material bias to our estimated ORs could arise due to loss to follow-up and missing data.

Since the time that we published our earlier analysis ( Lee & Fry, 2019) and our paper on general considerations relating to vaping as a possible gateway into cigarette smoking ( Lee *et al.*, 2018) a number of other authors have presented evidence from other prospective studies ( Bold *et al.*, 2018; Chien *et al.*, 2019; Kinnunen *et al.*, 2019; Morgenstern *et al.*, 2018; Pénzes *et al.*, 2018; Primack *et al.*, 2018; Treur *et al.*, 2018). The studies vary in the extent to which potential confounding variables have been adjusted for, with large OR estimates tending to be reported in studies with more limited control. Thus, a study in the Netherlands ( Treur *et al.*, 2018), which adjusted only for sex, age education and a single indicator of propensity to smoke, reported an OR of 11.90 (95% CI 3.36-42.11) for the relationship between ever use of e-cigarettes with nicotine and initiation of cigarette smoking during follow-up. Also, a study in the US ( Bold *et al.*, 2018), which adjusted only for demographic variables and use of other tobacco products, reported ORs of 7.08 (2.34-21.42) and 3.87 (1.86-2.06) depending on the follow-up period studied, while another US study ( Pénzes *et al.*, 2018), with limited control for confounding variables, reported an OR of 3.57 (1.96-6.45). Apart from a US study ( Primack *et al.*, 2018) ,which reported an OR of 6.8 (1.7-28.3), following adjustment for ten covariates independently associated with initiation of smoking, most of the other studies that appear to have better control for confounding gave lower estimates. These included a study in Taiwan ( Chien *et al.*, 2019), which reported an OR of 2.14 (1.66-2.75), a study in Germany ( Morgenstern *et al.*, 2018), which reported an OR of 2.18 (1.65-2.87) and a study in Finland ( Kinnunen *et al.*, 2019), which reported that adjustment reduced the OR from 11.52 (4.91-26.56) to 2.92 (1.09-7.85). Notably, a study in Great Britain ( East *et al.*, 2018) reported an OR of 11.89 (3.56-39.72) estimated using the usual logistic method, but a reduced value of 1.34 (1.05-1.72) using causal mediation analysis.

There have, by now, been a number of other papers that have studied the gateway effect in youths based on data from the PATH study. In our earlier paper ( Lee & Fry, 2019), we commented on an early publication ( Watkins *et al.*, 2018) based on data from Waves 1 and 2, noting that the list of variables adjusted for was quite restricted. Since then two other papers have been published based on Waves 1 and 2 ( Cheng *et al.*, 2019; Stanton *et al.*, 2019) and one based on data from Waves 1 to 3 ( Berry *et al.*, 2019). All of these studies took into account a more limited set of predictors than we had, and none used predictors assessed at a time before e-cigarette use was initiated. One study ( Berry *et al.*, 2019) found that prior e-cigarette use among youths aged 12 to 15 years was associated with 4.09 times (95% CI 2.97-5.63) the odds of ever cigarette use and with 2.75 times (95% CI 1.60-4.73) the odds of current e-cigarettes use compared with no prior tobacco use, while another ( Stanton *et al.*, 2019), based on the full youth sample, found that e-cigarette use was associated with 3.21 times (95% CI 1.95-5.45) the odds of ever cigarette smoking. Interestingly, the other study ( Cheng *et al.*, 2019), again based on the full youth sample, using a somewhat different approach, found that while the latent construct “common liability to use tobacco products” was a robust predictor for the onset of cigarette smoking, ever e-cigarette use was not a significant predictor, after controlling for this construct.

Generally our results are consistent with the literature in confirming that a substantial proportion, but not all, of the observed association between e-cigarette use and subsequent initiation of cigarette smoking can be explained by adjustment for factors linked to susceptibility to tobacco. However, large cohort studies with high quality, accurate, data on a wide range of predictive factors recorded at regular intervals will be needed to gain better insight into the magnitude of any true causal effect of vaping. The PATH study with its multiple Waves and comprehensive questionnaire should prove more and more useful in the future. It will also provide information on the relationship between e-cigarette use and continued smoking, it being possible that some of those classified as taking up smoking at Wave 3 in our analyses would have only briefly taken up smoking.

There are, in theory, various effects of e-cigarettes ( Lee *et al.*, 2018). Beneficial effects occur when individuals who would have continued to smoke take up vaping instead, and when vaping helps smokers to quit or reduce cigarette consumption. Adverse effects, apart from when vaping encourages individuals to start smoking, would occur if smokers who intended to quit switch instead to vaping, or if smokers add vaping to their usual consumption of cigarettes. When trying to estimate the health impact of e-cigarettes, one must consider all these effects.

A question of interest is the extent to which an estimated gateway effect could affect the total number of youths taking up cigarette smoking. As shown in the footnote to Table 1, analysis M1 was based on 409 youths who had taken up smoking between Waves 2 and 3, including 148 who had ever used e-products at Wave 2. The weighted unadjusted data are consistent with 36.5% of these being ever e-product users and with an OR for the gateway effect of 5.60. Assuming the adjusted OR based on adjustment for the variables making up the propensity score, this percentage would reduce to 23.0%. For the estimated ORs of 3.37 or 3.11, based on adjustment for the propensity score as a continuous variable or quintiles, this percentage would only change slightly, to 23.6% or 22.0%. This percentage would clearly vary according to the relative frequency of e-product use and cigarette smoking among youths, and the number of extra smokers would need to be set against the beneficial effects described in the previous paragraph.

By using data from three Waves of the PATH study, the analyses of the gateway effect reported here improve on those reported earlier ( Lee & Fry, 2019) based on the first two Waves by allowing potential confounding variables to be determined at a time before vaping started. Whereas the earlier analyses suggested that the adjustment for confounding explained about 80% of the unadjusted relationship between vaping and subsequent initiation of smoking, our current analyses suggest that adjustment explains only about 50%. This provides stronger evidence of a true effect of vaping, although doubt still remains about its true magnitude for reasons discussed.

## Data availability

### Underlying data

National Addiction & HIV Data Archive Program: Population Assessment of Tobacco and Health (PATH) Study [United States] Public-Use Files (ICPSR 36498). https://doi.org/10.3886/ICPSR36498.v9 ( United States Department of Health and Human Services (USDHHS), 2019).

The data are available under the Terms of Use as set out by ICPSR, which can be accessed when users start the process of downloading the data.

### Extended data

Open Science Framework: Further investigation of gateway effects using the PATH study https://doi.org/10.17605/OSF.IO/7ECQH ( Lee, 2020).

This project contains the following extended data files:

- Gateway paper for F1000 Research_Additional file.docx

Data are available under the terms of the Creative Commons Zero “No rights reserved” data waiver (CC0 1.0 Public domain dedication).
