# Supplementary material for: Further investigation of gateway effects using the PATH study
Source: F1000Res. 2020 Nov 9;9:607. Originally published 2020 Jun 15. [Version 2] doi: 10.12688/f1000research.24289.2 (PMC9020531; doi:10.12688/f1000research.24289.2)
Supplement: Peer review history

# Peer review: Reviewer response for version 2

**DOI:** 10.5256/f1000research.30516.r119714

**Published:** 2022-04-19

**Authors:** Arielle S. Selya

**Affiliations:** 1 Pinney Associates, Inc., Pittsburgh, PA, USA; 2 Sanford School of Medicine, University of South Dakota, Vermillion, SD, USA

This manuscript follows on previous work by the authors using the PATH study to address the question of whether e-cigarettes act as a "gateway" to cigarette smoking among youth. Previous work showed that adjusting for propensity for e-cigarette use accounted for about 80% of the association between e-cigarette use and subsequent cigarette smoking. However, that previous study suffered from possible overadjustment (i.e., if e-cigarette use affected the covariates at Wave 1), and the current study addresses this by examining another wave of PATH (i.e., covariates at W1, e-cigarette use at W2, and smoking outcome at W3). Analyses find that adjusting for shared risk (while avoiding overadjustment) explains about half of the association between e-cigarette use and smoking. This provides stronger evidence for a possible causal gateway mechanism than previously reported, though unaccounted-for confounding is still a limitation.

 Major comments:

- I agree with previous reviewer Shu Xu that covariate balance should be reported as part of good practice in using propensity score methods. E.g., standardized mean difference of < 0.2 or ratio of variances across groups between 0.2 and 2 (Kainz *et al.*, 2017 ^1^). Presenting covariate balance, even (or especially) if it does not achieve exact balance, is important to evaluate the degree to which any unadjusted-for bias still remains in the adjusted associations.
- What is the rationale for using PATH Waves 1-3 when Waves 4 and 5 (and for youth, 4.5) are available? Waves 1-3 are quite old now and the results may not generalize to the newer e-cigarette market.
- The authors have a paragraph discussing why there may be doubts about the magnitude of the suspected gateway association. Other reasons worth adding are (1) the issue of how well covariate balance was achieved (see the above point) and (2) that the presence of measurement error in certain variables (especially for latent constructs such as risk-seeking) can itself lead to spurious association (Westfall *et al.*, 2016 ^3^).

Minor comments:

- Consider giving an example to explain the concept of overadjustment in the Introduction -- this is central to the value of this study, but is not a well-known statistical issue in this field.
- Consider citing other studies supporting common liability, e.g. Sun *et al.* (2022 ^4^), Sokol *et al.* (2021 ^5^).

Is the work clearly and accurately presented and does it cite the current literature?

Partly

If applicable, is the statistical analysis and its interpretation appropriate?

Yes

Are all the source data underlying the results available to ensure full reproducibility?

Yes

Is the study design appropriate and is the work technically sound?

Partly

Are the conclusions drawn adequately supported by the results?

Partly

Are sufficient details of methods and analysis provided to allow replication by others?

Partly

Reviewer Expertise:

Tobacco use behavior, methodology, youth

I confirm that I have read this submission and believe that I have an appropriate level of expertise to confirm that it is of an acceptable scientific standard, however I have significant reservations, as outlined above.

## References

[1] : Improving Causal Inference: Recommendations for Covariate Selection and Balance in Propensity Score Methods. *Journal of the Society for Social Work and Research* .2017;8(2) : 10.1086/691464 279-303 10.1086/691464

[2] : Reporting of covariate selection and balance assessment in propensity score analysis is suboptimal: a systematic review. *J Clin Epidemiol* .2015;68(2) : 10.1016/j.jclinepi.2014.08.011 112-21 10.1016/j.jclinepi.2014.08.011 25433444

[3] : Statistically Controlling for Confounding Constructs Is Harder than You Think. *PLoS One* .2016;11(3) : 10.1371/journal.pone.0152719 e0152719 10.1371/journal.pone.0152719 27031707PMC4816570

[4] : Is Adolescent E-Cigarette Use Associated With Subsequent Smoking? A New Look. *Nicotine Tob Res* .2022;24(5) : 10.1093/ntr/ntab243 710-718 10.1093/ntr/ntab243 34897507PMC8962683

[5] : High School Seniors Who Used E-Cigarettes May Have Otherwise Been Cigarette Smokers: Evidence From Monitoring the Future (United States, 2009–2018). *Nicotine & Tobacco Research* .2021;23(11) : 10.1093/ntr/ntab102 1958-1961 10.1093/ntr/ntab102 PMC849646733991190

# Peer review: Reviewer response for version 2

**DOI:** 10.5256/f1000research.30516.r87806

**Published:** 2021-08-18

**Authors:** Jean Long

**Affiliations:** 1 Health Research Board, Dublin, Ireland

**Overall comments:**

 Overall, the authors need to complete an objective examination of the research based on findings rather than commentary or opinion. For example, the literature search, extraction, and referencing for the introduction requires reworking. I have not checked the referencing in the discussion but suggest the authors do so to ensure avoidance of error. The research question should use a PICO approach and then explain the rationale for doing the research (including explanation of confounding, residual confounding, and sensitivity analysis). The five-point statement has ethical and duty of care implications and should be removed. I have extensive comments on the introduction below. The methods as currently presented are not repeatable by other researchers and I have made extensive recommendations for rewriting (see below). The tables of results were for the most part clear, but the endnotes require work and I have made some recommendations for these and a small number of recommendations for the tables themselves. I have made little comment on the results text as I believe it requires a redrafting using a professional writer or editor. When the editor is finished with your work, please check the table numbers and numbers quoted in the text to ensure that they align, and they report the findings accurately. I also suggest the table headings are rewritten by the professional writer as they are difficult to understand on first reading. The discussion is confusing, contradictory, and incomplete when dealing with the excess risk contributed by e-cigarette use on initiation of smoking tobacco use or gateway effect, and requires complete rewriting supported by evidence-based research findings under the headings: main findings, comparison of findings with other research (beginning with your own, other primary studies (already there for the most part) and then systematic reviews), strengths and limitations of the research (including how you addressed your limitations), and finally implications for policy and practice (using best practice). I have provided detailed guidance below on the discussion. These steps would help a reader read and understand the paper. I could not recommend this paper for publication without a major rewrite and an improvement in objectivity, clarity, transparency, and an evidence-based analysis of the policy and research implications. I make more specific comments below.

**Is the work clearly and accurately presented and does it cite the current literature? No.**

 The systematic review literature is incomplete as there are more appropriate papers, with a particular emphasis on teenagers, that should be summarised. I recommend a thorough examination of the findings of the following papers and the inclusion of papers that are appropriate to the study population. I recommend that you quote the research findings and not commentary or opinion. I provide an example using the Khouja *et al.* paper on citation section.

 The current text reads as if the above named are the two papers, but I think this is not correct. As later we see text “the second paper”. Please amend.

 Is "it" in the sentence “It made a number of relevant points” Soneji *et al.* or Lee *et al.*? Please reference for the reader. I suggest that you quote the three points from Lee *et al.* (I suspect it is this paper) as it would be more accurate, and they should be expressed as theories as this is how they are phrased in Lee *et al.*

 Your five-point statement requires reconsideration, specifically “Any true gateway effect would only alter smoking prevalence modestly” implies that it is okay to allow some teenagers (mainly children) to start vaping and then move to smoke. My understanding of e-cigarettes and vaping is that the industry does not allow teenagers under 18 years to vape, as this point is repeated on a regular basis in the Irish media by those representing the industry.

 I suspect many people would disagree with you on the topic that it is okay to allow teenagers to start vaping and for over 20% to continue to smoke to help adults to stop smoking; there are some ethical and child protection issues here. In addition, there is no evidence to demonstrate taking-up vaping while giving-up smoking has reduced mortality, which is implied in your five-point statement. The longest study I can find on vaping is 24 months and it does not deal with mortality. I would also point out that vaping is not a necessary step to smoking cessation and that there are other equally or more effective interventions. In addition, there are some papers that show that vaping plus smoking is as harmful to the users health as smoking, and therefore, there is no benefit to dual use.

 With respect to the point "In youths in the US and UK in 2014–2016 smoking prevalence declined more rapidly than the preceding trend would predict, contrary to what might be expected if any large gateway effect existed." I put the opposite forward for consideration - it is possible that the decrease in smoking would have been even larger if e-cigarettes did not exist. This is an equally valid point and I think to date there is no evidence either way. In addition, I would add that one teenage child starting to vape and/or smoke is one too many.

 Please reference the six additional studies “six additional studies” as this is an important part of reproducibility.

 The authors should present the findings of Khouja *et al. *rather than using incomplete statements of commentary taken from the discussion. The point of your current paper is to determine if confounding has been adequately controlled for, not to justify the use of e-cigarettes. I suggest you present objective findings as they will speak for themselves.

 Here is what you should consider quoting from Khouja *et al.*, obviously you will need to summarise it:

 “The results of individual studies included in the main meta-analysis are: unadjusted [OR: 4.59, 95% CI: 3.60 to 5.85] and (adjusted OR: [2.92, 95% CI: 2.30 to 3.71]). Effect sizes (ORs) ranged from 2.46 to 12.31 (unadjusted) and [2.30 to 3.71] adjusted. All estimates were considered to show strong evidence of a positive association between e-cigarette use among non-smokers and later smoking in unadjusted analyses. Covariates included in the adjusted analyses varied on a study-by-study basis. After adjustment, effects in all but three studies remained strong.”

 With respect to “relying on self-reported measures”, I note, based on experience, that none of the existing cohort studies did biochemical verification of outcomes as they relied on the tried and tested questions about ever use, recent or last year use, and current or last 30 days use and these measures are accepted the world over for surveying the use of tobacco products, licit drugs, and illicit drugs. The most common measure of both e-cigarette and cigarette use was ‘ever use’ of either product, an indicator which has been critiqued by researchers in one paper [56], as it did not observe whether the teenagers used the product once in their young life, or if they used it regularly. ‘Past-30-day use’ has gotten the same censure. However, the use of these indicators has been justified, with a recent study by Birge *et al.* finding that over two-thirds of smokers who ever consumed a single puff of a tobacco cigarette during adolescence became, for a time, regular smokers [57].

 Taken from: O'Brien D, see citations.

 I would point out that you are relying on self-reported measures in all three studies, and if use of this data is inaccurate and misleading, a better use of time and resources might be to fund PATH to do independent biochemical verification of very current use and then run the analysis. However, I worked on the topics of alcohol, tobacco, and drugs for the past 20 years and in my experience, the findings will be that self-reported measures underestimate use indicating that the situation is more serious than demonstrated in surveys or cohort studies.

 The research question needs to be phrased specifying the population (in the PATH study), the intervention of interest, the comparator, and the outcomes measured. The population are teenagers living in the USA and this needs to be included in the analysis and discussion. The intervention of interest is the move from e-cigarettes to tobacco cigarette smoking. The outcome of interest is initiated tobacco cigarette smoking. Your current objective requires rewriting base on the above guidance and then you should go on to explain the rationale for your analysis explaining the limitations of the 2018 analysis.

**Is the study design appropriate and is the work technically sound? Partly.**

 I think the methods need to be rewritten as I had to read them three times to try and ascertain what the researchers did. Most readers will not do this, so a plain English and logical approach is required. The current paper requires the authors to read the 2018 paper before reading this paper and I do not think that this is good enough and it is very frustrating for a reader. Most people won’t do this.

 I recommend:

 When presenting a cohort study, the following facts need to be presented: title of the cohort study, objective of the cohort study, a description of the study population, total sample in WAVE 1 and the phase 1 study response rate, loss to follow-up for WAVE 2 as compared to WAVE 1, for WAVE 3 as compared to WAVE 1, for WAVE 3 as compared to WAVE 2, A list of covariates for each wave and any changes between waves should be presented. In addition, I would explicitly state the independent variable, dependent variable and covariates used in this analysis. Finally, the percentage of missing data for key variables needs to be reported. Response rates, loss to follow-up and missing data have implications for a valid and representative analysis, so I recommend that they are presented here and a judgement by the authors (Lee *et al.*) as to whether the quality of the PATH study is adequate for this analysis.

 I would then describe any selections and exclusions of data from the original cohort explaining why you did this and what are the implications for validity and representativeness.

 Then present the differences in how the PATH study variables were used in this study compared to the 2018 iteration. I would present them in a small table showing 2018 use and 2021 use as this would help the reader.

 I recommend you state the variables included in the propensity index. In your tables there are no participants aged 18 or over while in your text 18+ is mentioned but not clearly explained. Please explain the situation to the reader and in the results provide exact numbers and proportions located in the adult data.

 I recommend that you provide the reader with a description of a sensitivity analysis, explaining the rationale for doing the **four **sensitivity analyses, and the method you are using. You have a four bullet points, one for each sensitivity analysis, and I suggest you used these in a table with four columns (and five rows). The column titles are: Short title for sensitivity analysis; Sensitivity analysis descriptor; Rationale for each sensitivity analysis; Covariates for the sensitivity analysis. This would help with transparency and the reader.

 I recommend that you delete the term ‘main’ and ‘additional’ from the **five** analyses as they are confusing and main implies that there is only one principal analysis when there are three main analyses and two additional analyses. I recommend that you title them: Analysis 1, Analysis 2, Analysis 3, Analysis 4 and Analysis 5; please make the same changes in your table and text in the results section. The five analyses could be summarised in a table with four columns and six rows. The column titles could be: Analysis number; description of the analysis; difference with respect to the 2018 paper, and rationale for this difference. This would save on text but increase clarity for the reader. Then, please tell the reader how the four sensitivity analyses relate to the five analyses.

 I think the reader would want explanations of the ROELEE program, and the terms 'step function' and GLM package (considering what they are, why they are used, and how they are used).

**Are sufficient details of methods and analysis provided to allow replication by others? No.**

 The authors need to provide a much clearer description of what they did and why they did what they did to allow replication. In addition, the selectors (syntax) used to identify the data downloaded from PATH is required. They also need to provide the two syntax for cleaning and analysing the data. Apart from the publicly available data from PATH, I can’t access any of the supplementary information or additional files. This needs to be corrected by the journal.

**If applicable, is the statistical analysis and its interpretation appropriate? I think it may well be.**

 I found the results text difficult to read and understand and suggest that a professional editor is employed to rewrite the text and ensure that the text matches the tables. When the editor is finished with your work, please check the table numbers and numbers quoted in the text to ensure that they align. The tables are the best part of this report though I suggest some improvements for transparency and clarity. The endnotes require work and I have made some recommendations for these below and a small number of recommendations for the tables themselves. I also suggest the table headings are rewritten by the professional writer as they are difficult to understand on first reading.

 Table 1 requires the following corrections: percentage of total beside each N in column 3, exact age [add in years]; the base and numbers (%) for Ever been curious about smoking a cigarette, Think you will smoke a cigarette in the next year, Think you will try a cigarette soon and whether the base is yes or no and what the current confidence intervals represent; and any other categories in the three variables to make the 8058. I know you have small letters at the end that may entitle you to present incomplete data, but the explainer does not explain the data to me. It would be more correct and transparent to provide the full data for this variable and you can put your summary OR at the end and explain why and how you are using it in the end note and how this affects your regression analysis.

 In addition, Tables 2, 3, 4, 6, 7 require clearer end notes.

 Please revise end note text as follows. Example for table 2 “Between Waves 2 and 3 261/7367 (3.54%) of never users of e-products at Wave 2 started smoking, while 148/893 (16.57%) of ever users of e-cigarettes started smoking” as the existing text is unclear. Please ensure correct numbers for the end note to tables 3, 4, 6, 7.

 Please revise text as follows “For individuals who were 16 (n=) or 17(n=) at Wave 1, adult data (n=) were used to determine e-product use and cigarette smoking at later Waves; the percentage that were followed-up was %”; this increases transparency. Please ensure correct wave, age and numbers in tables 3, 4, 6, 7.

**Are the conclusions drawn adequately supported by the results? No.**

 The discussion is confusing, contradictory, and incomplete when dealing with the excess risk contributed by e-cigarette use on initiation of smoking tobacco use or gateway effect, and requires complete rewriting supported by evidence-based research findings under the headings: main findings, comparison of findings with other research (beginning with your own, other primary studies (already there) and then systematic reviews), strengths and limitations of the research (including how you addressed your limitations), and finally implications for policy and practice (using best practice).

 The first section of the discussion is not written in the usual format and is therefore confusing. I recommend that the authors should begin with a summary of their new analysis, then compare it to their previous analysis and explain why they have different results. The authors should then state clearly that there is a gateway effect and the minimum and maximise size of the gateway effect based on their best controlled analyses.

 I don't think the authors should speculate on what is an acceptable magnitude of effect as any 95% confidence intervals that do not include ‘1’ as this indicates a risk that e-cigarettes may introduce teenage children to take up smoking tobacco cigarettes. The authors need to consider their ethical responsibility and duty of care to children with respect to both e-cigarettes and tobacco cigarettes. Is between 22% and 24% of cigarette smoking in teenage children attributed to initiation of smoking tobacco cigarettes? Please explain the implication of this statistic, if e-cigarettes were removed from this cohort, I estimate that 34 teenagers in this study would not have smoked tobacco cigarettes. If we multiply this figure up to the USA’s teenage population, how many teenagers would not smoke?

 The current tone of the discussion reads as if the authors are trying to absolve the e-cigarette industry of taking responsibility for the consequences of their product and create doubt about any excess risk or risk that may be attributable to e-cigarettes by blaming the quality of the survey data that the authors themselves decided to use; this contradicts the authors' earlier statement that there is a gateway effect and raises questions as to why the authors did an analysis on inadequate data. I would suggest that if the survey data is inadequate then they should refrain from publishing the analysis. I recommend that the authors should list specific covariates missing form PATH (if covariates are actually missing) that may explain the unaccounted for or residual confounding and avoid generalities. In addition, the authors or industry could provide funding (that is untied) to PATH to do biochemical verification of very current use to test reliability. This would improve the quality of the PATH cohort study.

 There are no implications for research and policy presented here despite the admittance to a gateway effect and odds of initiating smoking that are above one. I recommend that the authors describe what actions should be taken by industry and national governments to stop children using e-cigarettes and smoking tobacco cigarettes. These actions should be evidence-based addressing regulation, price, limiting availability, and banning promotion as these are the types of actions that change behaviour. I would refrain from investing in education as the evidence indicates that this does not change behaviour.

 The abstract needs to be rewritten once the paper is rewritten.

Is the work clearly and accurately presented and does it cite the current literature?

No

If applicable, is the statistical analysis and its interpretation appropriate?

Partly

Are all the source data underlying the results available to ensure full reproducibility?

No

Is the study design appropriate and is the work technically sound?

Partly

Are the conclusions drawn adequately supported by the results?

No

Are sufficient details of methods and analysis provided to allow replication by others?

No

Reviewer Expertise:

Epidemiology, public health and substance use

I confirm that I have read this submission and believe that I have an appropriate level of expertise to state that I do not consider it to be of an acceptable scientific standard, for reasons outlined above.

## References

[1] : Patterns of E-Cigarette Use Among Youth and Young Adults: Review of the Impact of E-Cigarettes on Cigarette Smoking. *Nicotine & Tobacco Research* .2019;21(10) : 10.1093/ntr/nty103 1320-1330 10.1093/ntr/nty103 29788314

# Peer review: Reviewer response for version 2

**DOI:** 10.5256/f1000research.30516.r74455

**Published:** 2021-01-26

**Authors:** James Sargent

**Affiliations:** 1 C. Everett Koop Institute, Norris Cotton Cancer Center, Geisel School of Medicine at Dartmouth, Lebanon, NH, USA

I am satisfied with the authors' responses to my concerns. I find it of particular interest that, all else being equal (after propensity score adjustment), upwards of 20% of the youths taking up cigarette smoking can have their new onset smoking attributed to earlier use of e-cigarettes. I think this is a very important addition to the existing literature and speaks to the population-level importance of youth e-cigarette use.

Is the work clearly and accurately presented and does it cite the current literature?

Yes

If applicable, is the statistical analysis and its interpretation appropriate?

I cannot comment. A qualified statistician is required.

Are all the source data underlying the results available to ensure full reproducibility?

Yes

Is the study design appropriate and is the work technically sound?

Yes

Are the conclusions drawn adequately supported by the results?

Yes

Are sufficient details of methods and analysis provided to allow replication by others?

Yes

Reviewer Expertise:

Adolescent substance use.

I confirm that I have read this submission and believe that I have an appropriate level of expertise to confirm that it is of an acceptable scientific standard.

# Response

**Published:** 2021-01-26

**Authors:** Peter Lee

**Affiliations:** P.N.Lee Statistics and Computing Ltd, UK

I thank the reviewer for approving the revised version of the manuscript.

# Peer review: Reviewer response for version 2

**DOI:** 10.5256/f1000research.30516.r74456

**Published:** 2020-12-07

**Authors:** Shu Xu

**Affiliations:** 1 Department of Biostatistics, NYU School of Global Public Health, New York City, NY, USA; 2 New York University, New York, NY, USA

In the revised manuscript, authors have updated the manuscript with recent publications and also expand discussion based on the results of attrition analyses and current literature.

 In my previous review, I have pointed out that the manuscript is hard to follow because readers need to refer to two previously published articles to figure out research details. It is understandable that authors should avoid reporting overlapping materials from published articles, however, I recommend authors (1) focus on the current study and provide a complete and independent introduction of the CURRENT study (by summarizing instead of repeating the details), and (2) relocate the similarities and differences between earlier study and current study to the Discussion section. The introduction should emphasize why and what are new in the current study. For example, testing the potential heterogeneity among participants at various ages would be a contribution to the literature. Currently, the analyses were introduced in the Methods and Results session, however, it is unclear why these analyses were needed.

 Meanwhile, I would like to point out a few technical issues.

1. Discussion: A 13% attrition rate in a longitudinal study may not be trivial. In missing data literature, ^1^ Schafer considered a missing rate of 5% or less is ignorable. Bennett maintained that statistical analysis is likely to be biased when more than 10% of data are missing. Authors need to clearly state the assumption and implication of removing participants with missing data in a listwise fashion.
2. Reporting adjusted OR based on various adjustment approaches is not equivalent to achieving covariate balancing. Tables 2 – 4 may serve as sensitivity tests on how ORs would be impacted based on various adjustment of confounding. Given the manuscript focuses on the extent to which the exposure effect of e-cigarette can be explained by covariates, then it would be important to discuss (1) whether covariate balance has achieved in the data under study, and (2) what would be the possible consequence if any important covariate being ignored or not being measured in the current study.

Is the work clearly and accurately presented and does it cite the current literature?

Yes

If applicable, is the statistical analysis and its interpretation appropriate?

Partly

Are all the source data underlying the results available to ensure full reproducibility?

Yes

Is the study design appropriate and is the work technically sound?

Partly

Are the conclusions drawn adequately supported by the results?

Yes

Are sufficient details of methods and analysis provided to allow replication by others?

Partly

Reviewer Expertise:

Longitudinal data analysis, propensity score methods, missing data method, tobacco research.

I confirm that I have read this submission and believe that I have an appropriate level of expertise to confirm that it is of an acceptable scientific standard, however I have significant reservations, as outlined above.

## References

[1] : Principled missing data methods for researchers. *SpringerPlus* .2013;2(1) : 10.1186/2193-1801-2-222 10.1186/2193-1801-2-222 23853744PMC3701793

# Response

**Published:** 2021-01-29

**Authors:** Peter Lee

**Affiliations:** P.N.Lee Statistics and Computing Ltd, UK

**Reply to further comments made by Shu Xu**

 We thank the reviewer for their further comments, but were rather disappointed by their content, especially given that the other reviewer James Sargent considered that he could now approve version 2 of our paper based on the revisions we had made.

 In our paper Investigating gateway effects using the PATH study”, published in F1000 Research in 2019 and based on Waves 1 and 2 of that study, we concluded that confounding is a major factor explaining most of the observed gateway effect, but were concerned about the possibility of over-adjustment if taking up e-cigarettes had affected the values of some of the Wave 1 predictor variables considered.  We suggested in that paper that the possibility of over-adjustment could be avoided by relating initiation of cigarette smoking at Wave 3 to vaping at Wave 2, restricting attention to those who, at Wave 1, had never vaped, and using propensity indicators recorded at Wave 1 linked to uptake of e-cigarettes by Wave 2.  This is noted in the penultimate paragraph of the introduction of our current paper, and as we stated in the following paragraph the main objective was to conduct such analyses, though we also pointed out that we also included a variety of sensitivity and alternative analyses for reasons we described further on in the paper.

 Shu Xu recommends that we provide a “complete and independent” introduction of the current study and relocate the similarities and differences between the 2019 study and the current study to the discussion section.  We totally disagree with this idea – the current study arose out of suggestions made in the 2019 study, and is effectively an improved extension of it.  Accordingly it makes the paper much more understandable if, in the introduction, we start by summarizing what the 2019 study did and showed and make it absolutely clear that the current paper arose out of ideas proposed in the 2019 paper.  It would, in our view, be totally wrong to discuss similarities and differences between the 2019 paper and the current study in the discussion, as it would then not make clear to the reader at the outset the main objective of our paper.  Also, as so many of the methodological details were already described in the 2019 paper, there is really no need to give a greater description of what we did than is already in the methods.  We would expect the interested reader to look back at our 2019 paper if necessary.

 The first technical issue Shu Xu refers to relates to the 13% attrition rate which they argue may not be “trivial”.  We certainly did not regard it as trivial (describing it only as “not large”), since we conducted the various analyses summarized in the fourth paragraph of the discussion.  While we accept that there is always some possibility of bias due to attrition we feel that the arguments expressed in the last sentence of this paragraph leading to our conclusion that “it seems unlikely to us that any material bias to our estimated ORs could arise due to loss to follow-up and missing data” still hold. 

 The other technical issue Shu Xu refers to concerns their belief that the reporting of adjusted ORs based on various adjustment approaches “is not equivalent to achieving covariate balancing.”  One is trying to answer the question “does a never smoking individual who vapes have a different probability of taking up smoking, as compared to a never smoking individual who does not vape and who also has the same set of smoking predictors  as the one who vapes.”  As is generally the situation in epidemiological research, one cannot possibly ensure that one has achieved exact covariate balance and so we have used standard epidemiological techniques to deal with covariates.  However, though we have considered an extremely large number of variables (see Table 1 of the 2019 paper), certainly more than considered in most previous research on the gateway effect, we do already note (in the discussion in paragraph 3) that “there may be some relevant predictors or interactions of predictors not considered,” and our analyses already give results with or without adjustment for a range of predictors.  One cannot of course assess in practice the consequence of an important covariate not being measured in the current study, without knowing what the covariate is.  One can do hypothetical analysis for a mystery confounder with certain properties, but that is clearly beyond the scope of this paper.   There are already many theoretical statistical papers in the literature which investigate the bias that failure to consider relevant covariates might have, but we see no reason to refer to that here – we already consider this in our other 2019 paper “Considerations related to vaping as a possible gateway into cigarette smoking: an analytical review.”

 We prefer to leave our paper in the form it currently is. If the arguments that we express here convince Shu Xu to change their verdict to “Approved” we would of course be pleased.  If not, we will have to wait for the verdict of another peer reviewer before our paper can be sent to MedLine and other such databases.

# Peer review: Reviewer response for version 1

**DOI:** 10.5256/f1000research.26798.r71761

**Published:** 2020-10-13

**Authors:** Shu Xu

**Affiliations:** 1 Department of Biostatistics, NYU School of Global Public Health, New York City, NY, USA; 2 New York University, New York, NY, USA

The authors examined the association between youth prior e-cigarette use and increased risk of subsequent cigarette smoking using the Waves 1 – 3 data from the PATH study. This work is an extension of their previous studies which were published in Lee *et al.* (2018) and Lee and Fry (2019), the latter was based on the Waves 1 and 2 data from the PATH study. This study is interesting because the authors conducted three main analyses studying the association between e-cigarette use and subsequent cigarette smoking along with sensitivity analyses. This review emphasized the statistical methodology and results reporting. A few major concerns are below.

1. I feel the readability of this paper would be improved if authors could (1) focus on what is the limitation of the previous articles, (2) clearly state what are the new analyses about based on what has been done previously, and (3) state why versions of M1, M2, M3 were conducted and the logic behind them. The authors need to provide a full picture of the study design and analytical plan of the current study. In case some details are overlapped with previous articles when referred to the previous article, authors need to at least summarize the details instead of releasing no specific information.
2. The main concern of the previous study is about the possibility of “over-adjustment,” and the extent to which the association between prior e-cigarette use and subsequent cigarette initiation has been “over-adjusted.” It would be critical to evaluate whether covariate balance was sufficient when propensity scores had been considered in the current analyses. Without covariate balance, the results of the current study may be considered unreliable. Thus, detailed results such as (a) propensity score distribution by e-cigarette exposure groups and (b) comparison of the extent of covariate imbalance are desired.
3. In the Methods section, authors need to clearly state how the missing values were treated in analyses of the current study. This also involved how authors treated the missing values when selecting covariates of versions of M1, M2, and M3. The results of the current study could be misleading if only participants with complete data were considered.
4. It was unclear to me why to study the continuous age and grouped age and compare the difference. It seems like continuous age provided an exact measure however grouped age did not. Putting participants into categories is rarely defensible unless authors provide further justification. It is also unclear to me why only interactions with age (no other covariates, for example, race) were considered.

Minor concerns are below.

1. In tables, in addition to individuals who were 16-17 at Wave 1, adult data were used. Please clarify, for those who were 15-16 at Wave 1 (those who were 18+ at Wave 3), whether adult data were also used in this study?
2. The abstract was very confusing. It failed to provide an overview of the study. For example, a clear introduction of the methods and results of M1 and have been presented. This information regarding M2 and M3 were not clearly reported.

Is the work clearly and accurately presented and does it cite the current literature?

Yes

If applicable, is the statistical analysis and its interpretation appropriate?

Partly

Are all the source data underlying the results available to ensure full reproducibility?

Yes

Is the study design appropriate and is the work technically sound?

Partly

Are the conclusions drawn adequately supported by the results?

Yes

Are sufficient details of methods and analysis provided to allow replication by others?

Partly

Reviewer Expertise:

Longitudinal data analysis, propensity score methods, missing data method, tobacco research.

I confirm that I have read this submission and believe that I have an appropriate level of expertise to confirm that it is of an acceptable scientific standard, however I have significant reservations, as outlined above.

# Response

**Published:** 2020-10-28

**Authors:** Peter Lee

**Affiliations:** P.N.Lee Statistics and Computing Ltd, UK

**Reply to comments made by Shu Xu**

**We thank the reviewer for the time he has spent and the useful comments made.  Our replies to the points made are given in bold face type, making it clear where we have amended the original version of the paper.  Note that the changes made to the paper are also intended to answer the points made by James Sargent, the other reviewer.  We hope that our answers and the changes to the paper will allow the revision to be approved.**

 Approved With Reservations

 The authors examined the association between youth prior e-cigarette use and increased risk of subsequent cigarette smoking using the Waves 1 – 3 data from the PATH study. This work is an extension of their previous studies which were published in Lee *et al.* (2018) and Lee and Fry (2019), the latter was based on the Waves 1 and 2 data from the PATH study. This study is interesting because the authors conducted three main analyses studying the association between e-cigarette use and subsequent cigarette smoking along with sensitivity analyses. This review emphasized the statistical methodology and results reporting. A few major concerns are below.

- I feel the readability of this paper would be improved if authors could (1) focus on what is the limitation of the previous articles, (2) clearly state what are the new analyses about based on what has been done previously, and (3) state why versions of M1, M2, M3 were conducted and the logic behind them. The authors need to provide a full picture of the study design and analytical plan of the current study. In case some details are overlapped with previous articles when referred to the previous article, authors need to at least summarize the details instead of releasing no specific information.

**The three paragraphs of the discussion starting “Our second paper..” describe in some detail the analyses we had previously conducted using data from Waves 1 and 2 only, what the main results of these analyses were, and the fact that the estimates were open to the possibility of over-adjustment if taking up e-cigarettes had affected the values of some of the Wave 1 predictor variables considered.  It also makes it clear that our earlier paper described how this possibility could be avoided by using data from Waves 1, 2 and 3.  We have now amended the final paragraph of the discussion to make it clear that analysis M1 in the current paper was that envisaged in our earlier paper, and that this was the main objective of our work. In the methods section, there was already some comment on why we had conducted the other main analyses, the sensitivity analyses and the alternative analyses, but this has now been extended in various places to make it clearer.  Where details of our analyses are the same as those in our earlier analyses, it seems needlessly duplicative to repeat these details in the current paper, and is not the usual thing to do in such a situation.**

- The main concern of the previous study is about the possibility of “over-adjustment,” and the extent to which the association between prior e-cigarette use and subsequent cigarette initiation has been “over-adjusted.” It would be critical to evaluate whether covariate balance was sufficient when propensity scores had been considered in the current analyses. Without covariate balance, the results of the current study may be considered unreliable. Thus, detailed results such as (a) propensity score distribution by e-cigarette exposure groups and (b) comparison of the extent of covariate imbalance are desired. 

**Our latest paper has removed the possibility of over-adjustment in our previous work by the use of propensity indicators based on data recorded at Wave 1 in those who, at that time, had never vaped.  The reviewer questions whether covariate balance is sufficient after the propensity scores are taken into account.  This has been investigated in Tables 2, 3 and 4 for the three main analyses in turn by considering whether adjustment for the individual variables making up the propensity index materially affected the estimated gateway effect.  The effect was generally quite small, suggesting that reasonable balance had been achieved.  We think that including the additional material suggested by the reviewer would add little other than extra complexity.  We also note that our previous paper did not include such material and was approved by the reviewers who considered it.**

- In the Methods section, authors need to clearly state how the missing values were treated in analyses of the current study. This also involved how authors treated the missing values when selecting covariates of versions of M1, M2, and M3. The results of the current study could be misleading if only participants with complete data were considered.

**As we note in the first sentence of the methods section “Some aspects of the analyses described here are the same as those described earlier (Lee & Fry, 2019) are not presented again here.”  In that paper we made it clear that all the logistic regression analyses used “required individuals with complete data on all variables”, and that the various stages in developing propensity scores used “groups of conceptually-related variables, with missing values likely to be on the same individuals”.  We prefer not to repeat the description of this part of the methodology in the current paper.  However, in the new paragraph we have added into the discussion (starting “Other issues are...”), we have addressed your point that basing the analysis only on complete data might be misleading.  This point is similar to one raised by another reviewer.   We hope you find this satisfactory.**

- It was unclear to me why to study the continuous age and grouped age and compare the difference. It seems like continuous age provided an exact measure however grouped age did not. Putting participants into categories is rarely defensible unless authors provide further justification.

**As regards age, the 2019 paper we had published based only on Wave 1 and 2 subdivided individuals into ages 12-14 and 15-17 as the data were only available in that form.  Assuming that the Waves were conducted a year apart (which they approximately were) we could infer that those who were 12-14 at Wave 1 and 15-17 at Wave 2 were 14 at Wave 1 (and 15 at Wave 2), and that those who were 15-17 at Wave 1 and adults at Wave 2 were 17 at Wave 1 (and 18 at Wave 2). However we could not estimate the exact age of those who were 12, 13, 15 or 16 at Wave 1.  The position changed in the analyses using Wave 3 as well, as we could define those who were 12-14 throughout as 12 at Wave 1, those who were 12-14 at Waves 1 and 2 and 15-17 at Wave 2 as 13 at Wave 1 and so on.  While it would be preferable to use exact age throughout in some ways, here we were carrying out further analyses using the propensity index developed in the 2019 paper which included a term based on grouped age.  As the paper presents the main analyses using both grouped age and exact age, and the results were much the same, there is no real problem. **

- It is also unclear to me why only interactions with age (no other covariates, for example, race) were considered.  

**On the basis that age had a major effect on the rate of e-cigarette use and on uptake of smoking, we included interactions of age with the three predictors most strongly linked to the relevant gateway effect.  As this had essentially no effect on the estimates of the gateway effect, we felt that looking at further interactions would not be worthwhile.  Race was not a predictor that was included in the propensity index, so it seemed highly unlikely that including interactions with it would have had any major effect.  It would of course have been theoretically possible to consider many more predictors, including interactions of each predictor with each other predictor, higher order interactions, or quadratic or cubic terms in some predictors, but one has to stop somewhere.  However in the third paragraph of the discussion we have changed “there may be some relevant predictors not considered” to “there may be some relevant predictors or interactions of predictors not considered.”**

 Minor concerns are below.

- In tables, in addition to individuals who were 16-17 at Wave 1, adult data were used. Please clarify, for those who were 15-16 at Wave 1 (those who were 18+ at Wave 3), whether adult data were also used in this study?

**Those who were 17 at Wave 1 would have been 18 at Wave 2 so adult data would have been used.  Similarly, those who were 16 at Wave 1 would have been 18 at Wave 3 so adult data would again have been used.  However, those who were 15 at Wave 1 would not have been adults at Wave 3, so adult data were irrelevant. To avoid confusion we have changed age ranges like “16-17” to “16 or 17” in the various places they occurred in the paper.**

- The abstract was very confusing. It failed to provide an overview of the study. For example, a clear introduction of the methods and results of M1 and have been presented. This information regarding M2 and M3 were not clearly reported.

**We are constrained by the 300 word limit for the abstract, but have modified the abstract (particularly the methods section) to try to make things clearer.**

- Is the work clearly and accurately presented and does it cite the current literature?

**Yes**

- Is the study design appropriate and is the work technically sound?

**Partly**

- Are sufficient details of methods and analysis provided to allow replication by others?

**Partly**

- If applicable, is the statistical analysis and its interpretation appropriate?

**Partly**

- Are all the source data underlying the results available to ensure full reproducibility?

**Yes**

- Are the conclusions drawn adequately supported by the results?

**Yes**

 I confirm that I have read this submission and believe that I have an appropriate level of expertise to confirm that it is of an acceptable scientific standard, however I have significant reservations, as outlined above.

**We hope that we have answered the reviewer’s reservations adequately.**

# Peer review: Reviewer response for version 1

**DOI:** 10.5256/f1000research.26798.r66520

**Published:** 2020-07-22

**Authors:** James Sargent

**Affiliations:** 1 C. Everett Koop Institute, Norris Cotton Cancer Center, Geisel School of Medicine at Dartmouth, Lebanon, NH, USA

This is a thoughtful analysis of PATH data to determine an unbiased estimate of the relation between initial e-cigarette use among never cigarette smokers and subsequent cigarette smoking. I particularly like the idea of using W1 predictors of W2 e-cigarette onset to parcel out the over adjustment that could occur if these variables are assessed at the same time. I also liked the multitude of sensitivity analyses that showed it doesn't really matter, for example, how propensity scores are modeled. I see no major weaknesses. However I have a few suggestions.

1. It might not be unreasonable to have a statistician review the analysis.
2. This is a complete case analysis. Given that there are missing data for each individual variable and that there is loss to follow up, the authors need to convince us with some sort of sensitivity analysis that the results are not largely affected by attrition bias.
3. The literature review makes it seem like these are the only authors who have published on gateway effects using PATH data. They need to cite other PATH papers, point out weaknesses in them, and help us understand why this publication is worthy of attention. One worthy of particular attention used a propensity score analysis similar to these authors' W1-W2 analysis ^1^
4. One limitation not mentioned is that cigarette smoking onset does not make addicted cigarette smoker. This needs to be mentioned as a limitation. 
5. The authors miss some of the many studies that examined the relation between initial use of e-cigarettes and subsequent cigarette smoking. They could fill in that gap by mentioning and citing a meta-analysis conducted by Khouja in Tobacco Control that identified 17 prospective studies ^2^. It is worth comparing their best estimate with the combined estimates presented in that meta analysis.
6. Finally, given that there have been so many prospective studies, and all have pointed to a gateway effect, it seems reasonable to conclude that there is one, that is, that use of these devices independently increases risk for subsequent use of cigarettes. I realize that we could continue to quibble about the effect size, but this study does a good job of convincing us that the relative risk is real and that it is substantial, around 3. It seems like it might be an opportunity to also help us understand the population significance of the finding. The authors could do that with this population-based sample (which includes weights) by determining what proportion of the observed cigarette initiation is attributable to the gateway effect using attributable risk methods (risk difference as opposed to risk ratio). They could use the weights to determine the number of new cigarette initiators there were in the US that year attributable to e-cigarette exposure. This would be a real and novel contribution that would help investigators compare the public health consequences to youth with the public health consequences resulting from increased smoking cessation.

Is the work clearly and accurately presented and does it cite the current literature?

Yes

If applicable, is the statistical analysis and its interpretation appropriate?

I cannot comment. A qualified statistician is required.

Are all the source data underlying the results available to ensure full reproducibility?

Yes

Is the study design appropriate and is the work technically sound?

Yes

Are the conclusions drawn adequately supported by the results?

Yes

Are sufficient details of methods and analysis provided to allow replication by others?

Yes

Reviewer Expertise:

Adolescent substance use.

I confirm that I have read this submission and believe that I have an appropriate level of expertise to confirm that it is of an acceptable scientific standard, however I have significant reservations, as outlined above.

## References

[1] : Longitudinal e-Cigarette and Cigarette Use Among US Youth in the PATH Study (2013–2015). *JNCI: Journal of the National Cancer Institute* .2019;111(10) : 10.1093/jnci/djz006 1088-1096 10.1093/jnci/djz006 PMC679209530689915

[2] : Is e-cigarette use in non-smoking young adults associated with later smoking? A systematic review and meta-analysis. *Tob Control* .2020; 10.1136/tobaccocontrol-2019-055433 10.1136/tobaccocontrol-2019-055433 32156694PMC7803902

# Response

**Published:** 2020-10-28

**Authors:** Peter Lee

**Affiliations:** P.N.Lee Statistics and Computing Ltd, UK

**Reply to comments made by James Sargent**

**We thank the reviewer for the time he has spent and the useful comments made.  Our replies to the points made are given in bold face type, making it clear where we have amended the original version of the paper.  Note that the changes made to the paper are also intended to answer the points made by Shu Xu, the other reviewer.  We hope that our answers and the changes to the paper will allow the revision to be approved.**

 Approved With Reservations

 This is a thoughtful analysis of PATH data to determine an unbiased estimate of the relation between initial e-cigarette use among never cigarette smokers and subsequent cigarette smoking. I particularly like the idea of using W1 predictors of W2 e-cigarette onset to parcel out the over adjustment that could occur if these variables are assessed at the same time. I also liked the multitude of sensitivity analyses that showed it doesn't really matter, for example, how propensity scores are modeled. I see no major weaknesses. However I have a few suggestions.

- It might not be unreasonable to have a statistician review the analysis. 

**Both the authors of this paper are experienced statisticians, as is Shu Xu, the other reviewer.**

- This is a complete case analysis. Given that there are missing data for each individual variable and that there is loss to follow up, the authors need to convince us with some sort of sensitivity analysis that the results are not largely affected by attrition bias.

**We have added a new paragraph into the discussion, starting “Other issues are...” and hope this meets the reviewer’s point.**

- The literature review makes it seem like these are the only authors who have published on gateway effects using PATH data. They need to cite other PATH papers, point out weaknesses in them, and help us understand why this publication is worthy of attention. One worthy of particular attention used a propensity score analysis similar to these authors' W1-W2 analysis ^1^.

**We have added a new paragraph in the discussion, after the one referring to other studies on the gateway issue, to consider other studies using PATH data, including the Watkins study on which we had commented previously in our 2019 paper. **

- One limitation not mentioned is that cigarette smoking onset does not make addicted cigarette smoker. This needs to be mentioned as a limitation.

**At the end of the paragraph in the discussion starting “Generally our consistent” we have made the point that some of those recorded as taking up smoking at Wave 3 may only have taken it up for a short while, a limitation that can be answered better in analyses based also on data from later Waves.  ** 

- The authors miss some of the many studies that examined the relation between initial use of e-cigarettes and subsequent cigarette smoking. They could fill in that gap by mentioning and citing a meta-analysis conducted by Khouja in Tobacco Control that identified 17 prospective studies ^2^. It is worth comparing their best estimate with the combined estimates presented in that meta analysis.

**We are not sure why the reviewer thought we were not citing other studies.  The first paragraph of the introduction refers to the meta-analysis of Soneji et al. which considered nine studies, while the second paragraph of the introduction refers to our 2018 paper which includes a detailed commentary on 15 studies.  Also the fourth paragraph of the discussion refers to quite a number of recent studies.  However, we have now made it clear in the paragraph summarizing conclusions from our 2018 paper that it considered 15 cohort studies that have reported unadjusted and adjusted estimates of the gateway effect, nine considered in the 2017 meta-analysis by Soneji et al. and six additional studies.  We have also added a paragraph in the introduction mentioning the recent review by Khouja et al. that the reviewer referred to. ** 

- Finally, given that there have been so many prospective studies, and all have pointed to a gateway effect, it seems reasonable to conclude that there is one, that is, that use of these devices independently increases risk for subsequent use of cigarettes. I realize that we could continue to quibble about the effect size, but this study does a good job of convincing us that the relative risk is real and that it is substantial, around 3. It seems like it might be an opportunity to also help us understand the population significance of the finding. The authors could do that with this population-based sample (which includes weights) by determining what proportion of the observed cigarette initiation is attributable to the gateway effect using attributable risk methods (risk difference as opposed to risk ratio). They could use the weights to determine the number of new cigarette initiators there were in the US that year attributable to e-cigarette exposure. This would be a real and novel contribution that would help investigators compare the public health consequences to youth with the public health consequences resulting from increased smoking cessation.

**In order to illustrate the population effect we have added a new paragraph in the discussion (starting “A question of interest is..”) which estimates the percentage of new smokers associated with exposure to  e-cigarettes as about 23%.**

 Is the work clearly and accurately presented and does it cite the current literature?

**Yes**

- Is the study design appropriate and is the work technically sound?

**Yes**

- Are sufficient details of methods and analysis provided to allow replication by others?

**Yes**

- If applicable, is the statistical analysis and its interpretation appropriate?

I cannot comment. A qualified statistician is required. 

**See comment above.**

- Are all the source data underlying the results available to ensure full reproducibility?

**Yes**

- Are the conclusions drawn adequately supported by the results?

**Yes**

**References**

 1. Stanton C, Bansal-Travers M, Johnson A, Sharma E, et al.: Longitudinal e-Cigarette and Cigarette Use Among US Youth in the PATH Study (2013–2015). *JNCI: Journal of the National Cancer Institute*. 2019; **111** (10): 1088-1096 Publisher Full Text

 2. Khouja JN, Suddell SF, Peters SE, Taylor AE, et al.: Is e-cigarette use in non-smoking young adults associated with later smoking? A systematic review and meta-analysis. *Tob Control*. 2020. PubMed Abstract | Publisher Full Text

 I confirm that I have read this submission and believe that I have an appropriate level of expertise to confirm that it is of an acceptable scientific standard, however I have significant reservations, as outlined above.

**We hope that we have answered the reviewer’s reservations adequately.**
